# CcpA promotes *Staphylococcus aureus* virulence by directly controlling staphyloxanthin production

**DOI:** 10.1002/mlf2.70040

**Published:** 2025-12-25

**Authors:** Xian Chen, Huagang Peng, Xiancai Rao, Yi Yang, Keting Zhu, Zhen Hu, Shu Li, Xiaonan Huang, Feng Lin, Jianghong Wu, Weilong Shang, Renjie Zhou, Yifan Rao

**Affiliations:** ^1^ Department of Emergency Medicine Xinqiao Hospital, Army Medical University Chongqing China; ^2^ Department of Emergency Chinese People's Armed Police Force Characteristic Medical Center Tianjin China; ^3^ Key Laboratory of Microbial Engineering Under the Educational Committee in Chongqing, Department of Microbiology College of Basic Medical Sciences, Army Medical University Chongqing China

**Keywords:** catabolite control protein A, *crt* operon, pathogenicity, pigmentation, *Staphylococcus aureus*

## Abstract

*Staphylococcus aureus* is a notorious opportunistic pathogen with remarkable adaptability, enabling it to infect virtually every human tissue. Staphyloxanthin (STX), a critical virulence factor, contributes to *S. aureus* oxidative damage. However, the regulatory mechanism of STX production is incompletely understood. This study provides mechanistic insights into the role of catabolite control protein A (CcpA) in STX production. *ccpA* deletion considerably reduced STX yield in *S. aureus* strains with diverse genetic lineages. Western blot showed that CcpA inactivation did not alter SigB expression levels in *S. aureus*. Gene reporter and electrophoretic mobility shift assays revealed the direct control of CcpA on the expression of the *crtOPQMN* operon, which encodes enzymes for step‐wise STX biosynthesis. Moreover, CcpA deficiency remarkably impaired bacterial tolerance to H_2_O_2_‐mediated killing, decreased survival in whole‐blood treatment, and diminished persistence in macrophages. In mouse bacteremia and skin abscess models, CcpA was shown to enhance *S. aureus* virulence. Notably, inhibition of CcpA with Ag^+^ synergized with vancomycin to combat vancomycin‐intermediate *S. aureus* infections in vivo. Our findings establish CcpA as a SigB‐independent regulator of STX production, suggesting that targeting CcpA could be a promising antibiotic synergistic strategy for the management of multidrug‐resistant *S. aureus* infections.

## Impact statement

Staphyloxanthin (STX), a vital virulence factor, can protect *Staphylococcus aureus* from host immune clearance. The biosynthesis of STX in *S. aureus* is primarily controlled by the SigB–CrtOPQMN axis. However, SigB‐independent regulatory pathways for STX production are poorly characterized. This study reveals catabolite control protein A (CcpA) as a new regulator that directly controls CrtOPQMN expression to promote STX synthesis. Deletion of *ccpA* resulted in depigmentation of various *S. aureus* strains, while *ccpA* complementation considerably restored the golden pigmentation phenotypes. Overall, our research provides mechanistic insights into the regulation of STX production by CcpA in *S. aureus* and highlights a potential target for the development of therapeutic strategies to deal with *S. aureus* infections.

## INTRODUCTION

*Staphylococcus aureus* is a leading opportunistic pathogen that is responsible for clinical diseases, ranging from superficial skin infections to life‐threatening pneumonia, endocarditis, meningitis, and bacteremia^1^, ^2^. The prevalence of methicillin‐resistant *S. aureus* (MRSA) and vancomycin‐intermediate *S. aureus* (VISA) has further deteriorated the treatment of *S. aureus* infections^3^. The pronounced virulence of *S. aureus* arises from its ability to produce numerous virulence factors^4^, which are orchestrated by a sophisticated network of transcriptional regulators, including sigma factor B (SigB)^5^, SarA, and its homologs^6^, ^7^, and two‐component systems (TCSs), such as AgrAC^8^, AirSR^9^, GraRS^10^, and SaeRS^11^. These regulatory systems collectively govern the expression of virulence factors essential for *S. aureus* survival and pathogenicity. Deciphering these regulatory mechanisms and their associated virulence factors is crucial for developing innovative strategies to combat *S. aureus* infections.

Staphyloxanthin (STX), a carotenoid pigment produced by approximately 90% of *S. aureus* isolates^12^, is a key virulence factor for bacterial intracellular survival and adaptation to host immune responses^13^, ^14^. *S. aureus* strains deficient in STX show increased susceptibility to neutrophil‐mediated killing and reduced virulence in vivo^15^. In addition, STX‐deficient *S. aureus* strains decrease their burden during co‐infection with *Pseudomonas aeruginosa*, which highlights the essential role of STX in bacterial interactions^16^. STX synthesis is controlled by the *crtOPQMN* (*crt*) operon and *aldH* ^17^, ^18^. SigB is the major factor that governs the expression of *crt* operon^15^, ^19^. Other regulators, such as cold shock protein A (CspA, also known as MsaB)^17^, thioredoxin‐like oxidoreductase YjbH^20^, oxidative stress‐responsive regulator Spx^21^, and activator RsbU^22^, modulate the expression of genes involved in STX synthesis through a SigB‐dependent pathway. Moreover, some SigB‐independent pathways have been characterized for STX production. Catabolite control protein E negatively regulates the production of *S. aureus* STX by enhancing the transcription of *citB*, which encodes aconitase that controls bacterial tricarboxylic acid cycle^23^. The glycopeptide resistance‐associated TCS GraRS positively regulates STX production by targeting the *crt* operon in *S. aureus* strains^10^. Furthermore, exogenous components, such as tetrabromobisphenol A and carrot agar, can stimulate STX production, further augmenting the survival of *S. aureus* ^24^, ^25^. Therefore, the regulatory mechanisms of STX production are complicated and serve as new avenues for exploring the turning pathways of STX biosynthesis in *S. aureus*.

The catabolite control protein A (CcpA) is a key regulator of carbon catabolite repression and widely distributes in Gram‐positive bacteria, including *Bacillus subtillis*, *Streptococcus suis*, *Clostridium difficile*, and *Streptococcus pneumoniae* ^26^, ^27^. In protein level, the activity of CcpA is controlled by a phosphocarrier protein termed HPr‐Ser(P)‐46^27^. Under physiological conditions with abundant carbon sources, HPr undergoes phosphorylation at Ser46 to produce HPr‐Ser(P)‐46, which activates CcpA to repress certain genes involved in proline metabolism and tricarboxylic acid cycle of bacteria, such as *citA* and *citZ* ^27^. However, the active CcpA can also upregulate genes involved in cellular glycolysis and acetate metabolism by binding to the *cre* (catabolite‐responsive element) sites located on the gene promoter regions^27^, ^28^. CcpA is a powerful regulator that governs a large proportion of genome‐encoded genes in the host bacteria^26^. Peng et al.^29^ reported that *ccpA* deletion in VISA strain XN108 changed the expression levels of 893 genes compared with the wild‐type strain. In addition to metabolic regulation, CcpA influences diverse biological functions of bacteria, including biofilm formation, virulence‐factor production, stress response, drug resistance, and bacterial sporulation^30^, ^31^. These versatile functions highlight the importance of CcpA in bacterial adaptation and infection.

In this study, we observed that CcpA inactivation decreased STX production in VISA strain XN108, MRSA USA300, and methicillin‐susceptible *S. aureus* (MSSA) Newman. CcpA knockout markedly impaired bacterial antioxidant defenses, as evidenced by reduced survival under H_2_O_2_ stress. Molecular analyses revealed that CcpA directly binds to the *crt* operon promoter to activate STX biosynthesis, independent of SigB signaling. Phenotypically, *ccpA* deletion decreased *S. aureus* survival in whole‐blood treatment and macrophage phagocytosis, and attenuated bacterial virulence. Notably, inhibition of CcpA using Ag^+^ reduced STX production and synergized with vancomycin (VAN) to treat VISA infections in vivo. Overall, these findings establish CcpA as a vital regulator of STX‐mediated virulence via a SigB‐independent pathway, proposing CcpA targeting as a therapeutic strategy against *S. aureus* infections.

## RESULTS AND DISCUSSION

### *ccpA* deletion reduces STX production in *S. aureus*

One of the most important biological characteristics of *S. aureus* is its ability to produce the membrane‐bound golden pigment called STX, which enables the bacterium to form golden colonies on agar plates^9^, ^20^. Studies have shown that STX is a critical virulence factor with powerful antioxidant activity^32^. Disruption of STX synthesis leads to accelerated clearance of *S. aureus* by the host immune system, considerably increasing the survival rate of the infected animals^23^. Selvaraj et al.^33^ utilized the plant extract carvacrol to suppress STX synthesis of the MRSA strain ATCC33591; this suppression increased the survival rate of greater wax moths from 40% to 90% compared with the strain without carvacrol treatment.

**Figure 1:**
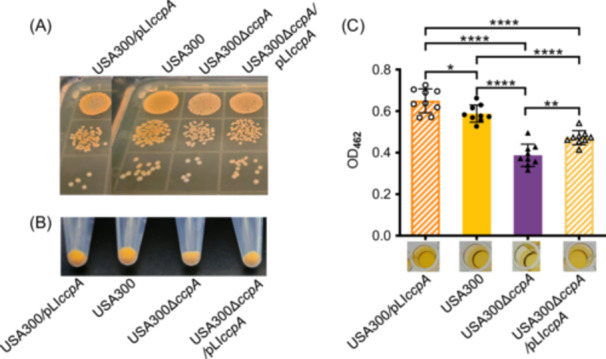
Deletion of *ccpA* reduces staphyloxanthin (STX) production in MRSA USA300. (A) Colonies of *Staphylococcus aureus* USA300 and its derivatives. (B) Bacterial pellets of USA300 and its derivatives. (C) STX yield of USA300 and its derivatives. Bacterial colonies of each strain were cultured with BHI broth and cells were pelleted. STX was extracted with methanol and quantified by detecting OD_462_ values. The experiments were repeated three times, and data are shown as mean ± SD (*n* = 9). Statistical significance was analyzed by one‐way ANOVA. *p < 0.05; **p < 0.01; ****p < 0.0001.

To study *S. aureus* resistance, we knocked out the *ccpA* gene in a VISA strain XN108 and observed reduced STX production (Figure S1). To confirm this observation, we deleted the *ccpA* gene in MRSA strain USA300 (Figure S2A). Decreased STX yield was achieved in USA300∆*ccpA* (Figure 1A), while *ccpA* complementation restored the pigmentation of USA300∆*ccpA*/pLI*ccpA*. Moreover, the strains of interest were cultured in BHI broth overnight, and the cell pellets of the USA300∆*ccpA* presented a lighter yellow color compared with those of the wild‐type USA300. In contrast, overexpression of *ccpA* in USA300, i.e., USA300/pLI*ccpA*, resulted in an even more pronounced yellow color of the cell pellets relative to the wild‐type USA300 (Figure 1B). Furthermore, bacterial STX was extracted using methanol, and the optical density at 462 nm (OD_462_) measurement revealed that the synthesis of STX in USA300∆*ccpA* was significantly reduced compared with that in USA300 (*p* < 0.0001), while *ccpA* complementation increased STX production of USA300∆*ccpA*/pLI*ccpA* relative to USA300∆*ccpA* (*p* < 0.01; Figure 1C). In contrast, *ccpA* overexpression resulted in remarkably increased STX production of USA300/pLI*ccpA* compared with USA300.

Next, the MSSA strain Newman was used to further investigate the effect of CcpA on STX synthesis, and its *ccpA* knockout strain was generated (Figure S2B). As expected, Newman∆*ccpA* showed reduced STX production compared with the wild‐type and *ccpA* overexpressed strains, and complementation with the intact *ccpA* gene restored the STX yield similar to the wild‐type Newman (Figure S3). Overall, these findings suggest that CcpA may be involved in the regulation of STX synthesis in *S. aureus* strains of diverse genetic lineages.

### CcpA deficiency reduces *S. aureus* tolerance to H_2_O_2_‐mediated killing

Reactive oxygen species (ROS) are a category of highly reactive molecules generated when ground‐state oxygen molecules acquire electrons^34^. Normal physiological processes produce small amounts of ROS that are essential for metabolic and regulatory functions. However, the excessive amounts of exogenous ROS generated by the NADPH oxidase system within the phagocyte phagosome induce oxidative stress, leading to oxidative damage, especially H_2_O_2_ and superoxide (O_2_^−^) damage^35^. Therefore, various systems exist in organisms for the production and removal of ROS to uphold intracellular redox balance dynamically^35^, ^36^. As a critical virulence factor of *S. aureus*, STX exerts its effect predominantly through antioxidant and cytotoxic actions^37^. The synthesis of STX in *S. aureus* is governed by the *crt* operon and *aldH*, which encode required enzymes to catalyze the sequential synthesis of STX from farnesyl diphosphate, a substrate produced by the bacterial tricarboxylic acid cycle^23^. The *crt* operon is highly conserved among *S. aureus* isolates, and its expression level directly determines the strength of the pigment phenotype^9^, ^19^. Deletion of the *crt* operon in USA300 and Newman completely eliminated STX production (Figure S4).

**Figure 2:**
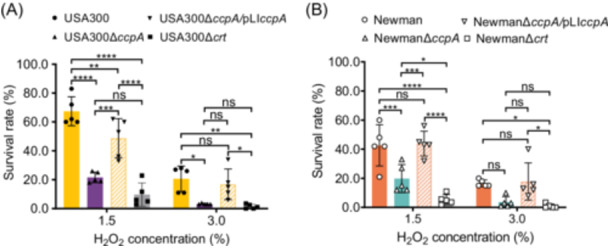
CcpA enhances the tolerance of *S. aureus* to H_2_O_2_‐mediated killing. The *S. aureus* strains USA300 and its derivatives (A) and Newman and its derivatives (B) were cultured at 37°C for 24 h with shaking. Approximately 2 × 10^8^ CFU of *S. aureus* were incubated in sterile PBS with 1.5% or 3.0% (v/v) H_2_O_2_ at 37°C for 1 h. The percent survival was calculated as described in the Materials and Methods section. The data represent the mean ± SD (*n* = 5). Statistical significance was analyzed by two‐way ANOVA. *p < 0.05; **p < 0.01; ***p < 0.001; ****p < 0.0001; ns, no significance.

Redox reactions are strongly intertwined with energy metabolism^35^, ^36^, ^38^. CcpA, a key regulatory molecule for carbon metabolism in *S. aureus* ^31^, promotes STX production (Figures 1 and S3). In this regard, we next explored whether *ccpA* knockout affected the tolerance of *S. aureus* to H_2_O_2_ inactivation. To do so, we prepared 1.5% (v/v) and 3.0% concentrations of a H_2_O_2_ solution to treat *S. aureus* strain USA300 and its derivatives. Colony counting assay revealed that the survival rates after treatment with 1.5% H_2_O_2_ for 1 h were 67.4%, 21.6%, 48.8%, and 9.7% for USA300, USA300∆*ccpA*, USA300∆*ccpA*/pLI*ccpA*, and USA300∆*crt*, respectively (Figure 2A). After treatment with 3.0% H_2_O_2_ for 1 h, the survival rate of USA300∆*ccpA* decreased to 3.2%, which was significantly lower than that of the wild‐type USA300 (20.6%, *p* < 0.05). By contrast, *ccpA* complementation led restoration of the survival rate of USA300∆*ccpA*/pLI*ccpA* to 16.8% (Figure 2A). In the case of strain Newman, 1.5% H_2_O_2_ challenge reduced the survival rate of Newman∆*ccpA* to less than half (19.8%) of the wild‐type Newman (42.6%). Complementation with *ccpA* restored the survival rate of Newman∆*ccpA*/pLI*ccpA* to 43.8%, comparable to that of the wild‐type strain (Figure 2B). On treatment with 3.0% H_2_O_2_ for 1 h, Newman, Newman∆*ccpA*, and Newman∆*ccpA*/pLI*ccpA* demonstrated comparable survival rates, while Newman∆*crt* showed the lowest survival rate. Overall, these findings show that CcpA inactivation markedly decreases the protection of *S. aureus* from H_2_O_2_‐mediated killing.

### CcpA enhances STX production by directly regulating *crt* operon expression

**Figure 3:**
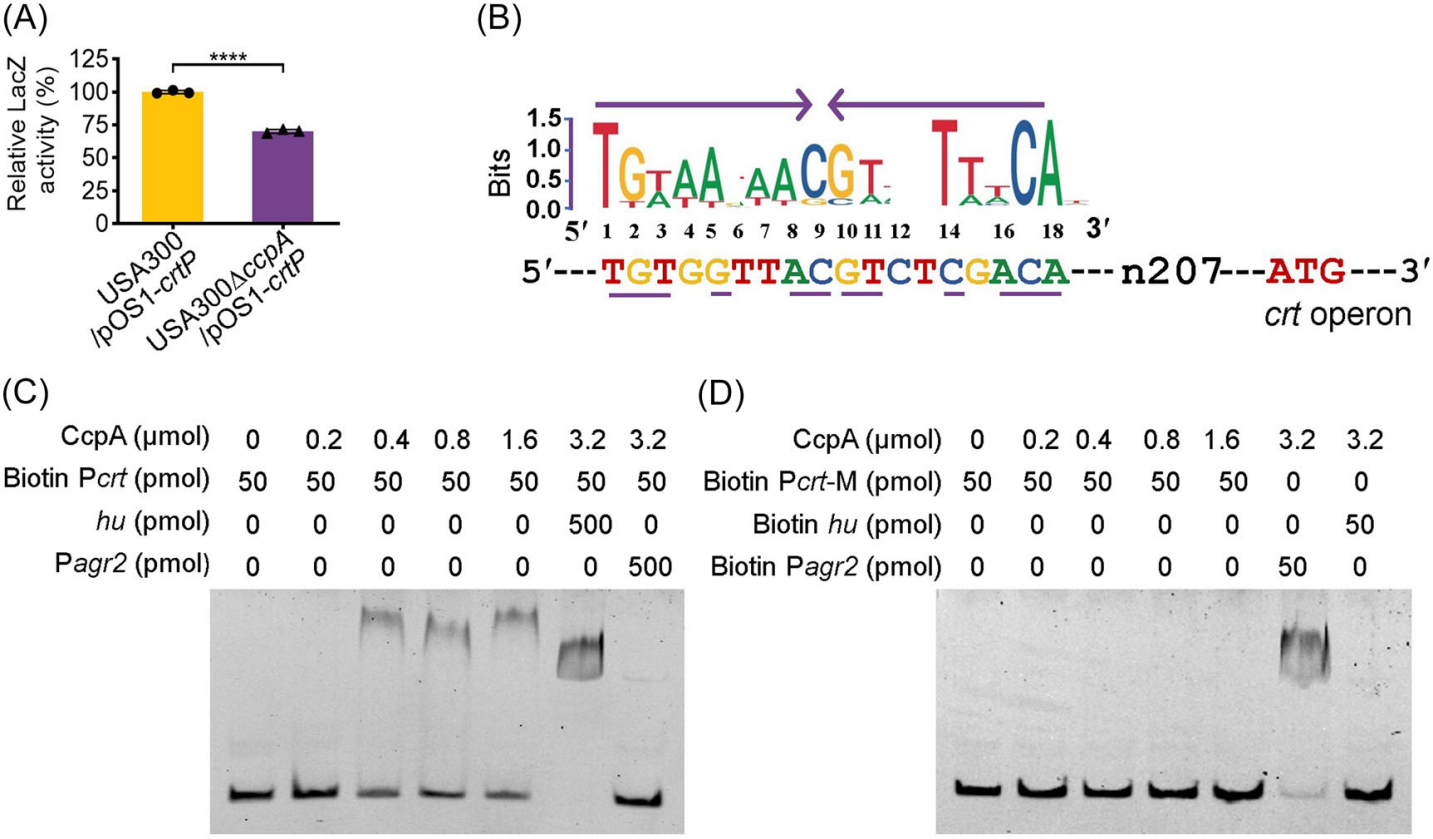
CcpA directly binds to the *crt* promoter region to activate STX biosynthesis. (A) β‐Galactosidase reporter assay. After transformation of the pOS1‐*crt*P reporter plasmid into USA300 and USA300Δ*ccpA*, the LacZ activity was detected. The data are presented as mean ± SD (*n* = 3). Statistical significance was calculated using Student's *t*‐test. ****p < 0.0001. (B) CcpA‐binding site predicted in the *crt* promoter regions. (C, D) Electrophoretic mobility shift assay with the DNA probe of the *crt* promoter region (P*crt*) (C) and the mutant P*crt* (P*crt‐*M) (D). The *agr2* promoter DNA fragment (P*agr2*) and the nonrelated *hu* gene promoter DNA probes were used as positive and negative controls, respectively.

Given that USA300∆*ccpA* and USA300∆*crt* showed similar tendency in response to H_2_O_2_ stress (Figure 2), we next sought to investigate whether a regulatory relationship exists between CcpA and the *crt* operon. To achieve this, a reporter vector (pOS1‐*crt*P) was generated (Table S1), and the β‐galactosidase assay showed that LacZ activity significantly decreased in USA300∆*ccpA*/pOS1‐*crt*P compared with that in USA300/pOS1‐*crt*P (*p* < 0.0001, Figure 3A). This result suggested that CcpA could affect the activity of the *crt* promoter in *S. aureus*. Subsequently, a real‐time quantitative polymerase chain reaction (RT‐qPCR) was performed to examine the expression‐level changes in *crtO*, *crtP, crtQ*, *crtM*, and *crtN* genes in strains USA300 and USA300∆*ccpA*. As shown in Figure S5A,B, the expression levels of *crt* genes decreased in USA300∆*ccpA* compared with those in USA300 cultured in vitro or infected in vivo. These data demonstrate that CcpA regulates *crt* operon expression in *S. aureus*.

The expression of the *crt* operon is mainly controlled by alternative SigB, and other regulators, such as RsbU, CspA, YjbH, and Spx, transcriptionally regulate *crt* operon expression in a SigB‐dependent manner^9^, ^20^, ^21^, ^32^. However, Western blot showed that the SigB protein levels in USA300 and USA300∆*ccpA* were comparable (Figure S5C), suggesting that the regulation of STX synthesis by CcpA could be independent of SigB. In general, CcpA regulates its downstream genes through a *cre* element^39^. The promoter regions of the *crt* operon were analyzed to determine if CcpA can directly regulate *crt* expression, and a possible CcpA binding site was predicted (Figure 3B). Then, His‐tagged CcpA proteins were engineered (Figure S6A), and a probe with the predicted CcpA binding site and its mutant were designed (Table S2). Electrophoretic mobility shift assay (EMSA) showed that CcpA could bind to the putative CcpA binding site‐carrying *crt* promoter DNA fragment (Figure 3C), but not to the fragment with a mutated CcpA binding site (Figure 3D). Meanwhile, the gray scale values of the free‐probe bands decreased gradually with an increase in CcpA proteins (Figure S6B). The binding was outcompeted by the addition of 10‐fold CcpA‐specific unlabeled P*agr2* promoter DNA. However, the 10‐fold addition of unrelated *hu* promoter DNA did not inhibit binding (Figure 3C). These results suggest that CcpA might increase STX production in *S. aureus* by directly controlling the expression of the *crt* operon and a SigB‐independent mechanism may be involved in CcpA regulation of STX production in *S. aureus*.

To further investigate the effect of STX on the binding between CcpA and the P*crt* probe, we extracted STX from *S. aureus* USA300. The addition of 0, 0.1, 0.5, 1.0, and 5.0 mg of STX did not affect the binding of CcpA to P*crt* probes (Figure S7A). To determine whether the exogenous addition of STX during bacterial culture could influence the expression level of the *ccpA* gene, *S. aureus* strains USA300 and USA300∆*crt* were cultured with or without STX treatment (1.0 mg/ml) at 37°C for 6 h. Total RNA was extracted, and RT‐qPCR revealed that the treatment of *S. aureus* with STX did not significantly change *ccpA* expression levels in bacteria during their growth in vitro (Figure S7B,C). Moreover, comparable *ccpA* expression levels were presented in USA300 cultured in vitro (BHI or DMEM) or infected in mouse livers at 6 hours post infection (hpi) (Figure S7D). Collectively, these data show that the exogenous addition of STX does not affect *ccpA* expression and the regulation of CcpA on the *crt* operon.

### Knockout of *ccpA* increases bacterial clearance by whole blood and RAW264.7 macrophages

**Figure 4:**
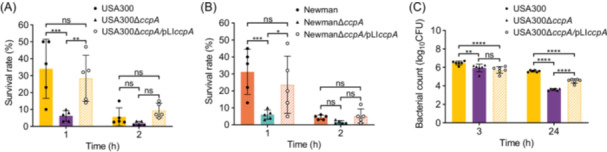
CcpA increases the survival *S. aureus* during whole‐blood‐mediated killing and macrophage phagocytosis. (A, B) Survival rate of *S. aureus* strains USA300 and its derivatives (A) and Newman and its derivatives (B) after whole‐blood‐mediated killing. The strains were cultured in BHI broth overnight. On the following day, bacterial cells were collected, inoculated into mouse whole blood, and incubated at 37°C for 1 or 2 h (*n* = 5). (C) Bacterial count of USA300 and its derivatives at 3 and 24 h post‐phagocytosis by RAW264.7 macrophages (*n* = 6). The data are presented as mean ± SD. The statistical significance was calculated by two‐way ANOVA. *p < 0.05; **p < 0.01; ***p < 0.001; ****p < 0.0001; ns, no significance.

During infection, *S. aureus* is exposed to complex conditions that threaten its survival^40^. Immune cells, such as neutrophiles that circulate in the blood and macrophages that distribute throughout the body, eliminate invading bacteria by oxidative killing^32^, ^41^. Thus, we sought to determine if the regulation of STX production by CcpA contributes to *S. aureus* tolerance to host immune clearance. Approximately 5 × 10^6^ colony‐forming unit (CFU) in 50 μl of phosphate‐buffered saline (PBS) of *S. aureus* USA300, USA300∆*ccpA*, and USA300∆*ccpA*/pLI*ccpA* were individually mixed with 450 μl of whole‐blood sample collected from Sprague–Dawley mice. After culturing at 37°C for 1 or 2 h, bacterial survival rates were measured by colony counting. As shown in Figure 4A, the survival rate of USA300∆*ccpA* (6.2%) after 1 h of incubation considerably decreased compared with the survival rates of the wild‐type strain (34.0%) and USA300∆*ccpA*/pLI*ccpA* (28.4%). After 2 h of treatment, *S. aureus* strains USA300, USA300∆*ccpA*, and USA300∆*ccpA*/pLI*ccpA* showed survival rates of 5.6%, 1.8%, and 9.4%, respectively. The bacterial survival rate of the Newman strain and its derivatives showed a similar tendency (Figure 4B). These results demonstrate that CcpA inactivation reduces the survival of *S. aureus* during whole‐blood‐mediated killing.

The mechanisms underlying whole‐blood‐mediated killing of *S. aureus* may be complicated. To assess the role of CcpA, we next determined if CcpA inactivation affects *S. aureus* survival in macrophages. RAW264.7 macrophages were infected with *S. aureus* USA300 or its isogenic derivatives at a multiplicity of infection (MOI) of 10. At 2 hpi, extracellular bacteria were eradicated by the addition of lysostaphin and gentamicin and incubated for another 1 h, and this time point was designated as 3 hpi. Intracellular bacterial counts were determined by plating on BHI agar. As shown in Figures 4C and S8A, USA300∆*ccpA* presented significantly reduced survival within macrophages compared with wild‐type USA300 at both 3 hpi (*p* < 0.01) and 24 hpi (*p* < 0.0001). These findings show that *ccpA* deletion promotes the swift clearance of *S. aureus* within macrophages. However, even with increased bacterial counts at 24 hpi, *ccpA* complementation only partially restored the survival rate of USA300∆*ccpA*/pLI*ccpA* in RAW264.7 macrophages compared with that of USA300∆*ccpA* (*p* < 0.0001, Figure 4C). The reason may be attributed to the compensatory strain carrying a pLI*ccpA* plasmid, which exacerbates the growth burden of USA300∆*ccpA*/pLI*ccpA* ^42^. We therefore constructed growth curves of USA300, USA300∆*ccpA*, and USA300∆*ccpA*/pLI*ccpA*. The results demonstrated that the complementation strain USA300∆*ccpA*/pLI*ccpA* showed a significantly lower growth rate than the wild‐type strain USA300 at both 2 and 3 h of culture (*p* < 0.001), but no notable difference was observed in comparison with the growth rate of USA300∆*ccpA* (Figure S8B). Thus, we speculate that the impaired growth of USA300∆*ccpA*/pLI*ccpA* during early infection might contribute to the diminished survival in macrophages. Overall, these data indicate that CcpA promotes *S. aureus* tolerance to both whole‐blood‐ and macrophage‐mediated oxidative clearance by upregulating the antioxidant STX production.

### CcpA‐regulated STX production contributes to *S. aureus* pathogenicity

*S. aureus* produces hemolysins that play critical roles in bacterial pathogenicity^43^. As one of the most important virulence factors, *S. aureus* hemolysin can target red blood cells, monocytes, platelets, and epithelial and endothelial cells^43^, ^44^. To investigate the impact of *ccpA* deletion on bacterial virulence, we first assessed the hemolytic activity of USA300 and its derivatives in vitro. As shown in Figure S9, the hemolytic activity of USA300∆*ccpA* considerably reduced compared with that of the wild‐type USA300, *ccpA‐*overexpressed USA300/pLI*ccpA*, and USA300∆*crt*. Complementation of the *ccpA* gene partially recovered hemolytic activity of USA300∆*ccpA*/pLI*ccpA* compared with the USA300∆*ccpA*. These data show that CcpA affects hemolytic activity in *S. aureus*. Seidl et al.^45^ reported that *ccpA* deletion reduces virulence‐factor expression in *S. aureus* by downregulating RNAIII, a regulatory noncoding RNA molecule of the accessory gene regulatory (*agr*) system that controls the expression of hemolysins such as Hla. Our results further confirm the effect of CcpA on *S. aureus* hemolytic activity as previously reported^46^.

**Figure 5:**
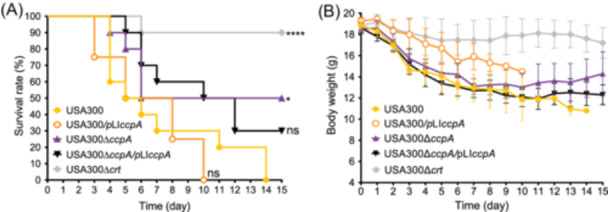
Survival and body weight variation of mice infected with *S. aureus* USA300 and its derivatives. (A) Mouse survival analysis. (B) Body weight change in mice after challenged with *S. aureus*. BALB/c mice (*n* = 10 per group) were challenged with 1 × 10^7^ CFU of USA300 or its derivatives through a tail‐vein injection, and the survival rate and body weight were calculated. The statistical significance was calculated using the log‐rank test. *p < 0.05; ****p < 0.0001; ns, no significance.

To further determine if the reduced bacterial survival within immune environments alters the virulence of USA300∆*ccpA*, we infected BALB/c mice with 1 × 10^7^ CFU of bacteria through a tail‐vein injection. Mouse mortality and body weight were monitored and recorded for 15 days. The results indicated that 50% of the USA300‐infected mice died at 5 days postinfection (dpi), and 100% of the mice died at 14 dpi (Figure 5A). In contrast, the USA300∆*ccpA*‐challenged mice presented a 50% survival rate, which was significantly higher than that of the USA300‐infected mice (*p* < 0.05). However, a 90% survival rate was achieved in mice infected with USA300∆*crt*, markedly higher than that of the WT‐infected mice (*p* < 0.0001). The body weights of all infected mice gradually decreased within 15 dpi, and USA300∆*crt‐*infected mice presented the slowest decrement in body weight among all groups; USA300∆*ccpA*‐infected mice showed higher body weights compared with both the wild‐type and complemented challenged ones (Figure 5B). Given the similar hemolytic activities of USA300∆*crt* and USA300 (Figure S9), the variable virulence presented by the isogenic strains could be ascribed to the STX production. Overall, these results show that CcpA inactivation considerably reduces the virulence of *S. aureus*.

**Figure 6:**
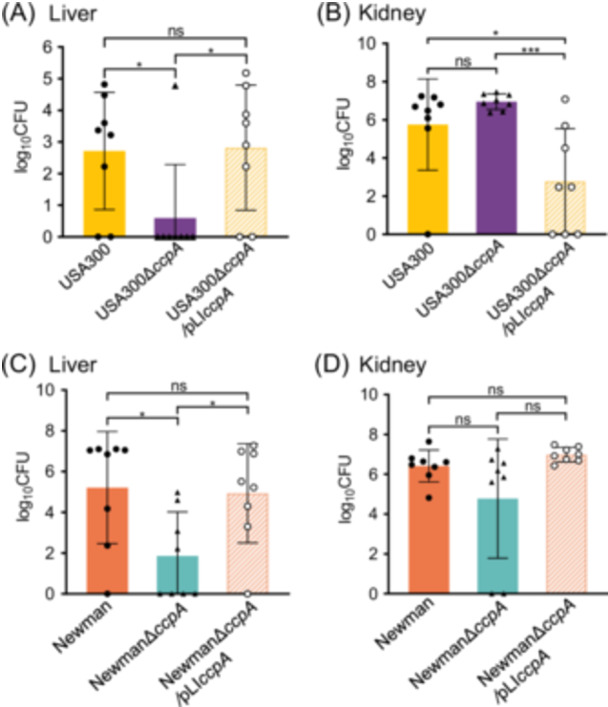
CcpA promotes *S. aureus* colonization in mouse organs. (A, B) Bacterial number of *S. aureus* strains USA300 and its derivatives in the livers (A) and kidneys (B) of the mice (*n* = 8) at 5 dpi. (C, D) Bacterial number of *S. aureus* strains Newman and its derivatives in the livers (C) and kidneys (D) of the mice (*n* = 8) at 5 dpi. The data are presented as the mean ± SD. The statistical significance was calculated by one‐way ANOVA. *p < 0.05; ***p < 0.001; ns, no significance.

Next, a bloodstream infection model was established in BALB/c mice by a tail‐vein injection of 1 × 10^6^ CFU bacteria to explore the change in *S. aureus* virulence after *ccpA* deletion. At 5 dpi, the animals were killed, and their organs, including the livers and kidneys, were obtained and homogenized for bacterial counting. The results showed that the bacterial burden of USA300∆*ccpA* in the liver was markedly lower than that of USA300 and USA300∆*ccpA*/pLI*ccpA* (Figure 6A). However, the bacterial load of USA300∆*ccpA* in the kidneys displayed no evident difference compared with that of the wild‐type USA300. Meanwhile, the complementation strain USA300∆*ccpA*/pLI*ccpA* showed the lowest colonization level in the kidneys at 5 dpi (Figure 6B). This phenomenon may be ascribed to the individual sensitivity discrepancy in mice or the randomly distributed bacteria in mouse organs after infection^6^, ^43^.

Given the distinct characteristics of the strain used, we intravenously injected BALB/c mice with 1 × 10^6^ CFU of Newman, Newman∆*ccpA*, or Newman∆*ccpA*/pLI*ccpA* to repeat the experiments. As shown in Figure 6C, the bacterial load of Newman∆*ccpA* in the liver was significantly lower than that of Newman and Newman∆*ccpA*/pLI*ccpA* (*p* < 0.05), while complementation with *ccpA* restored the load of Newman∆*ccpA*/pLI*ccpA* in the liver relative to that of the wild‐type Newman. However, in the kidneys, the bacterial burden of Newman∆*ccpA* was comparable to that of Newman and the complementation strain (Figure 6D). The lack of a significant difference in bacterial loads between USA300∆*ccpA* and wild‐type Newman may be ascribed to interorgan variability. Studies have revealed that even with identical inoculation dosage, organ‐specific bacterial colonization levels showed substantial variation^6^, ^43^. Collectively, these findings suggest that CcpA inactivation decreases the pathogenicity of *S. aureus*.

**Figure 7:**
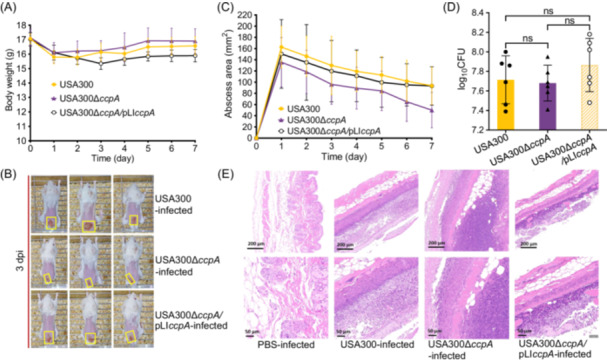
CcpA promotes *S. aureus* skin infection. (A) Body weight variation of BALB/c mice after a subcutaneous infection of 1 × 10^7^ CFU of USA300 or its derivatives. (B) Representative abscesses in mice at 3 dpi. The abscess size for each animal is indicated by a yellow box. (C) The abscess‐area change of mice during infection. (D) Bacterial burdens in mouse skin abscesses (*n* = 6). The statistical significance was calculated by one‐way ANOVA. ns, no significance. (E) Histological observation. The skin of infected mice was obtained at 7 dpi. Histological sections were prepared, stained with hematoxylin–eosin (H&E), and observed under a microscope. Scale bar, 200 or 50 µm.

Aside from causing deep‐tissue infections, *S. aureus* also frequently causes superficial skin infections characterized by surface abscesses^6^, ^43^, ^47^. In this study, a skin abscess mouse model was developed using female BALB/c mice. After the removal of back hairs, 1 × 10^7^ CFU of bacteria were administered via a subcutaneous injection. As shown in Figure 7A, no significant difference in body weight was observed among all the mouse groups at 7 dpi. However, the degree of skin ulceration in the USA300∆*ccpA*‐infected mice was lower than that in the USA300‐ and USA300∆*ccpA*/pLI*ccpA*‐challenged mice at 3 dpi (Figure 7B). Moreover, the abscess area caused by USA300∆*ccpA* was smaller than that caused by USA300 and USA300∆*ccpA*/pLI*ccpA* at 7 dpi (Figure 7C). Bacterial counting revealed comparable bacterial loads of USA300, USA300∆*ccpA*, and USA300∆*ccpA*/pLI*ccpA* in the skins (Figure 7D). Hematoxylin–eosin (H&E) staining showed that compared with the skins infected with USA300 and USA300∆*ccpA*/pLI*ccpA*, the skins infected with USA300∆*ccpA* showed lower levels of inflammatory infiltration (Figure 7E). Altogether, these data indicate that the regulation of STX production by CcpA contributes to the virulence of *S. aureus*. Thus, CcpA inhibition may be a promising strategy to control *S. aureus* infections.

### Inhibition of CcpA using Ag^+^ reduces STX production and synergizes with VAN to combat VISA infections

It has been reported that the pyocyanin pigments produced by *Pseudomonas aeruginosa* contribute to bacterial antibiotic resistance^48^. Therefore, we evaluated whether STX affects the susceptibility of *S. aureus* to conventional antibiotic treatment. As presented in Table S3, the addition of STX did not alter the susceptibilities of USA300 and its isogenic derivatives to most tested antibiotics, except for clindamycin. Strains USA300 and USA300∆*ccpA* were resistant to clindamycin, while USA300∆*crt* presented a susceptible phenotype with a minimum inhibitory concentration (MIC) of 0.094 μg/ml (Figure S10A). However, the exogenous addition of STX did not restore the clindamycin resistance of USA300∆*crt*. Moreover, Newman strains showed susceptibility to clindamycin regardless of STX addition (Figure S10B). This observation suggests that the impact of STX on *S. aureus* resistance can be strain‐ or antibiotic‐specific. The endogenous STX production may contribute to the resistance of USA300 to clindamycin, while the mechanism is unclear and needs further investigation.

**Figure 8:**
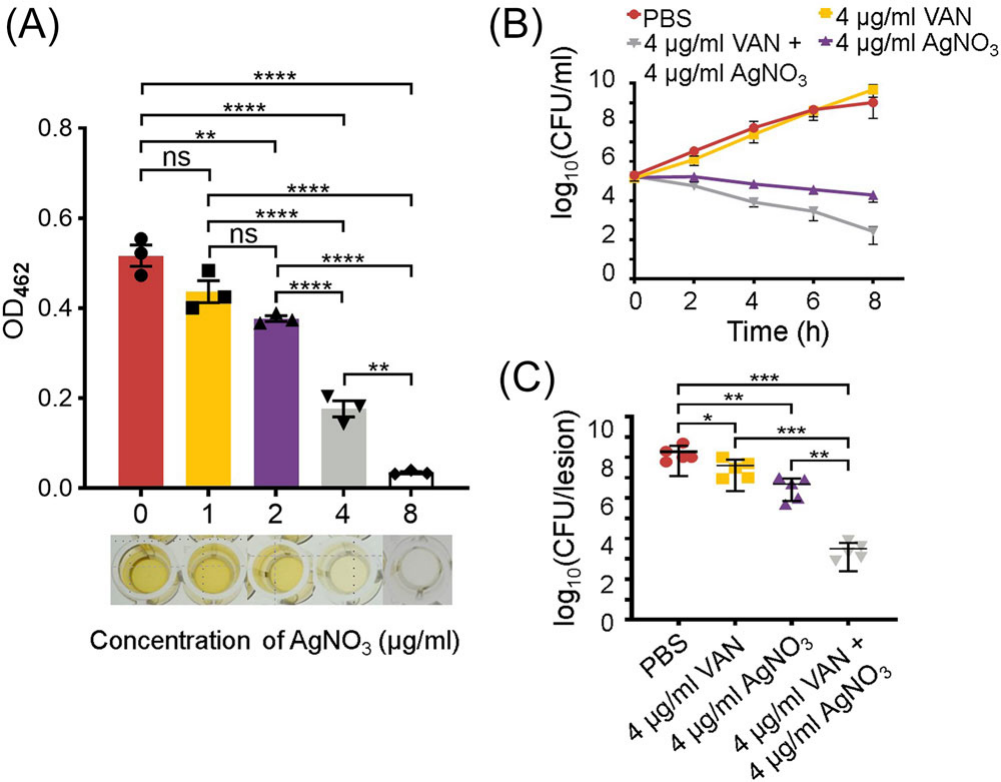
Silver ions inhibit STX production and exert synergistic effects with VAN against VISA infection. (A) VISA XN108 reduced its STX yield after treatment with Ag^+^. Bacteria were cultured in BHI broth with or without AgNO_3_. STX was extracted from bacterial pellets with methanol, and the OD_462_ value was determined. The statistical difference was determined by one‐way ANOVA. **p < 0.01; ****p < 0.0001; ns, no statistical significance. (B) Growth curve of VISA strain XN108 cultivated in BHI media with or without AgNO_3_ and/or VAN. Data shown are the mean ± SD from three independent experiments. (C) Bacterial load in the skin abscesses caused by XN108 after treatment with vehicle‐control ointment carrying VAN and/or AgNO_3_ twice a day. The log_10_CFU values are presented as the mean ± SD. The statistical difference was determined using the Mann–Whitney *U‐*test. *p < 0.05; **p < 0.01; ***p < 0.001. VAN, vancomycin; VISA, vancomycin‐intermediate *S. aureus*.

Given that the addition of STX alone did not remarkably alter the susceptibility of *S. aureus* to most clinically relevant antibiotics, we therefore focused on the synergistic effect of STX inhibition on antibiotic therapy. VAN is considered as a last line of drug for the treatment of serious MRSA infections^43^, thus, VAN was selected for initial investigation in combination with STX inhibition. Ag^+^ has been identified as an inhibitor of CcpA in *S. aureus* ^49^. When we determined the activity of Ag^+^ alone against VISA strain XN108, an MIC of 8 µg/ml AgNO_3_ was achieved. The production of STX in XN108 decreased gradually after treatment with 1–8 µg/ml (1/8–1 MIC) of AgNO_3_ (Figure 8A). Half MIC of VAN (4 µg/ml) did not inhibit XN108 growth in vitro, while half MIC of AgNO_3_ had an evident effect on XN108 growth (Figure 8B). The combination of 4 µg/ml VAN and AgNO_3_ showed a remarkable VISA‐killing effect. These data show that Ag^+^ and VAN exert synergistic anti‐VISA effects in vitro.

Next, we determined if Ag^+^ synergized with VAN improves VISA clearance in vivo. A skin abscess model was established by subcutaneously injecting 1 × 10^9^ CFU of XN108 (in 100 μl) into BALB/c mice. Then, the infected mouse skins were treated twice a day with sterile cream containing 4 µg/ml VAN and/or 4 µg/ml AgNO_3_. At 72 hpi, the mice were killed, and the abscess skins were excised and homogenized for bacterial counting. As shown in Figure 8C, VAN and AgNO_3_ at half MICs remarkably decreased the VISA load in the skin abscess. The combination of VAN and Ag^+^ showed a stronger VISA clearance effect compared with VAN and Ag^+^ treatment alone (*p* < 0.01). Overall, these data indicate that Ag^+^‐mediated inhibition of CcpA synergizes with VAN in treating VISA infections.

In conclusion, we identified CcpA as a SigB‐independent regulator that controls STX production, thereby enhancing *S. aureus* virulence. CcpA*‐*deficient strains showed a substantial reduction in STX production and decreased tolerance to H_2_O_2_‐ and whole‐blood‐mediated killing, while complementation with *ccpA* effectively restored the corresponding phenotypes. The LacZ reporter assay and EMSA revealed that CcpA directly regulates the *crt* operon, which encodes enzymes for STX biosynthesis in *S. aureus*. Inactivation of CcpA remarkably decreased the virulence of *S. aureus*, and CcpA inhibition with Ag^+^ considerably decreased STX production and synergized with VAN against *S. aureus* infections. Collectively, this study provides mechanistic insights into CcpA‐mediated regulation of STX production and highlights that targeting CcpA could be a promising synergistic strategy for treating *S. aureus* infections.

## MATERIALS AND METHODS

### Bacterial strains and plasmids

The bacterial strains and plasmids used in this study are presented in Table S1. The MRSA strain USA300 (ATCC‐BAA‐1556) was provided by Dr. Min Li (Shanghai Jiaotong University, China), MSSA strain Newman (NCTC 8178) was a gift from Dr. Lu Yu (Jilin University, China), and the VISA strain XN108 was isolated from a burn patient suffering from an acute *S. aureus* infection^50^. The pLI50 plasmid was selected for the gene complemented experiment, the pOS1 vector was applied to generate the reporter, pET‐28a was adopted to produce recombinant CcpA proteins, and the *Escherichia coli*–*S. aureus* shuttle plasmid pBT2 was utilized in gene deletion. *S. aureus* strains were cultivated in brain–heart infusion [BHI or BHI agar (Oxoid)]. *E. coli* strains were cultured in Luria–Bertani (LB) medium or on LB agar (Oxoid). The medium supplemented with chloramphenicol (Cm, 20 µg/ml), ampicillin (AMP, 100 µg/ml), or kanamycin (Kan, 50 µg/ml) was prepared as required^51^.

### Construction of gene deletion and complementation strains

The mutant with the *ccpA* deletion was generated using the homologous recombination strategy as previously described^43^. Briefly, the upstream and downstream DNA fragments of the *ccpA* gene were obtained by PCR from the DNA template of *S. aureus* USA300 using primers pBT2‐*ccpA*‐up‐F/pBT2‐*ccpA*‐up‐R and pBT2‐*ccpA*‐down‐F/pBT2‐*ccpA*‐down‐R (Table S2), and cloned into the pBT2 vector with the HiFi DNA Assembly Master Mix (NEBuilder). The resultant plasmid pBT2Δ*ccpA* was electroporated into *E. coli* DH5α competent cells and then spread onto an LB agar plate containing 100 μg/ml of AMP. After culture at 37°C overnight, the AMP‐resistant colonies were picked up and cultivated in LB broth with AMP at 37°C overnight. The plasmid was extracted and electroporated into *S. aureus* RN4220 for modification, followed by electroporation into *S. aureus* USA300. The mutant strain, termed as USA300Δ*ccpA*, was screened out as Cm‐sensitive colonies grown on BHI agar plates and verified by PCR assay and DNA sequencing. Similar strategies were performed to construct NewmanΔ*ccpA*, XN108Δ*ccpA*, USA300Δ*crt*, and NewmanΔ*crt* using the primer pairs listed in Table S2.

For the generation of *ccpA* complementation strains, the intact *ccpA* gene with its native promoter was obtained from USA300 genomic DNA by PCR with primer pairs pLI50‐*ccpA*‐F and pLI50‐*ccpA*‐R, and cloned into the pLI50 vector to generate pLI*ccpA*. Afterward, the pLI*ccpA* was transformed into *S. aureus* RN4220, and then electroporated into *S. aureus* USA300Δ*ccpA* to obtain the USA300Δ*ccpA*/pLI*ccpA* strain. A similar method was used to construct strain NewmanΔ*ccpA*/pLI*ccpA*.

### Detection of STX

The STX concentration in *S. aureus* was determined as previously described^25^. In brief, the overnight culture of bacteria was centrifuged at 13,000*g* for 1 min. The bacterial pellets were washed two times with sterilized water, resuspended in 200 μl of methanol, and heated at 55°C for 3 min. After centrifugation, the supernatant was transferred to a 96‐well plate and the OD_462_ value was determined using a Bio‐Tek microplate reader (Thermo Fisher Scientific). The experiment was conducted three times.

A carotenoid assay kit (LEAGENE) was used to quantify the carotenoid content in the extracted STX samples. The spectrophotometer (Thermo Scientific, USA) was preheated for at least 30 min and calibrated using the carotenoid assay buffer. The OD_440_ value of each STX sample was measured, and the carotenoid concentration was calculated using the formula N (mg/g) = [OD_440_/(250 × 1)] × *V* × *N* × 1000/*W*, where *V* represents the volume of the STX sample (ml), *N* denotes the dilution factor, *W* is the wet or dry weight of the sample (g), 250 is the empirical extinction coefficient for carotenoids (l/g/cm), 1000 is the conversion factor from grams to milligrams, and 1 corresponds to the cuvette pathlength (cm).

### H_2_O_2_‐mediated killing assay

The H_2_O_2_‐mediated killing assay was conducted as previously described^9^. Briefly, the overnight culture of *S. aureus* was centrifuged at 13,000*g* for 1 min, and bacterial pellets were washed twice with PBS (pH 7.2). Approximately 2 × 10^8^ CFU of bacterial cells were incubated at 37°C in a 1.5% or 3.0% (v/v) H_2_O_2_‐carrying PBS solution for 60 min. Then, the culture was serially diluted and plated onto agar plates. After culture at 37°C overnight, the colonies were counted. The survival rate (%) was calculated using the formula (number of CFU_final_/number of CFU_input_) × 100.

### β‐Galactosidase assay

The DNA fragment of *crt* operon promoter regions was amplified by PCR from the *S. aureus* USA300 DNA template using the primers presented in Table S2. The PCR products were digested using *Eco*RI and *Bam*HI restriction enzymes, purified, and subcloned into the pOS1 plasmid, which contains a promoterless *LacZ* gene^52^. The resulting plasmid (pOS1‐*crt*P) was transformed into *E. coli* DH5α and characterized by DNA sequencing. After transformation into *S. aureus* RN4220 for restrictive modification, the pOS1‐*crt*P was then transformed into *S. aureus* USA300 and USA300Δ*ccpA*. After incubation at 37°C overnight, the culture was 1:100 diluted with fresh broth and cultured at 37°C with shaking for 7 h to obtain an OD_600_ of 1.2. After culture, the bacterial cells were harvested by centrifugation at 5000*g* for 10 min, washed twice with PBS, and resuspended in AB buffer (100 μl; 100 mM KH_2_PO_4_, 100 mM NaCl, pH 7.0). The β‐galactosidase assay was conducted as described^43^. In brief, bacterial cells were lysed with lysostaphin (Sigma‐Aldrich) at a final concentration of 1 mg/ml by incubating at 37°C for 15 min. Then, 900 μl of ABT (AB buffer with 0.1% Triton X‐100) was added, and 50 μl of the sample was mixed with 10 μl of 4‐methylumbelliferyl β‐d‐galactoside (4 mg/ml, Sigma‐Aldrich) and incubated in the dark at 37°C for 1 h. Finally, 20 μl of the mixture was combined with 180 μl of ABT solution, and the reaction was monitored at 445 nm. All samples were analyzed in triplicate.

### RT‐qPCR

*S. aureus* cells cultured in vitro or infected in vivo (*n* = 3) were prepared as previously described^53^. The total RNA of *S. aureus* was extracted using an RNAprep Pure Cell/Bacteria Kit (Tiangen Biotech Co.). The corresponding cDNA was synthesized using a RevertAid First Strand cDNA Synthesis Kit (Thermo Fisher Scientific). Afterward, qPCR was conducted using an SsoAdvanced™ Universal SYBR®Green Supermix kit (Bio‐Rad) to determine the expression levels of each target gene. The *gyrB* gene was included as a reference. The primer pairs for each *crt* gene are detailed in Table S2.

### Western blot

The protein levels of SigB in USA300 and USA300∆*ccpA* were detected by Western blot. Briefly, *S. aureus* was cultured overnight in BHI broth at 37°C. The cells were harvested from a 2 ml culture, washed twice with PBS, and resuspended in 1 ml of PBS on ice. Then, 2 g of zirconia‐silica beads were added and the bacterial cells were disrupted with Minibeadbeater (Biospec, USA) for three cycles (5 min for each). The concentration of cellular proteins was measured using a Bradford protein assay kit (Beyotime Biotechnology). *S. aureus* proteins were separated by 12% (m/v) sodium dodecyl sulfate polyacrylamide gel electrophoresis (SDS‐PAGE) and transferred to a polyvinylidene difluoride membrane (GE Healthcare). The membrane was blocked at 37°C for 1 h using TBST buffer [50 mM Tris, 138 mM NaCl, 2.7 mM KCl, pH 8.0, 0.05% (v/v) Tween 20] supplemented with 5% (m/v) nonfat milk (Boster). The primary murine antibodies against SigB (provided by Prof. Hao Zeng, Army Medical University) and secondary sheep anti‐mouse antibodies conjugated with horseradish peroxidase (Boster) were utilized for detection. The protein bands of interest were visualized using an enhanced chemiluminescence apparatus (Amersham Pharmacia Biotech).

### Protein expression and purification

The full‐length *ccpA* gene was amplified from *S. aureus* USA300 genomic DNA using the primers listed in Table S2. Then, the PCR fragment was cloned into the expression vector pET28a (Novagen, Germany) to obtain the recombinant plasmid pET28‐*ccpA*. After transformation into *E. coli* BL21 (DE3) competent cells, the engineered bacteria were grown in LB at 37°C to an OD_600_ of 0.6 and induced with 1 mM isopropyl‐β‐d‐thiogalactoside (IPTG) at 25°C for 20 h. Afterward, the cells were harvested and resuspended in buffer A (50 mM NaH_2_PO_4_, 300 mM NaCl, 10 mM imidazole, pH 8.0). Bacterial cells were lysed by high‐pressure homogenization, and the solution was centrifuged at 10,000*g* at 4°C for 30 min. The supernatant was applied to a 5 ml HisTrap HP column (Cytiva), and the recombinant CcpA proteins were purified using an AKTA purifier (GE Healthcare) with buffer B (50 mM NaH_2_PO_4_, 300 mM NaCl, 250 mM imidazole, pH 8.0). The proteins were pooled and dialyzed against buffer C (300 mM NaCl, 50 mM NaH_2_PO_4_, pH 8.0) at 4°C for 4 h. After concentration by a HisTrap HP column, CcpA proteins were collected and subjected to gel filtration with a HiLoad 16/600 Superdex 75‐pg column (Cytiva). The purified CcpA proteins concentrated to 1 mg/ml were stored at −80°C.

### EMSA

EMSA was conducted as previously described^43^. Briefly, the biotin‐labeled DNA fragments of *crt* promoter regions containing the putative CcpA‐binding site (P*crt*) were amplified from *S. aureus* USA300 genomic DNA using the primers Crt‐EMSA‐5‐Biotin/Crt‐EMSA‐3 and Crt‐EMSA‐5/Crt‐EMSA‐3 (Table S2). The biotin‐labeled DNA mutants (P*crt*M, changing the “A” or “T” in the putative CcpA‐binding site to “C” or “G”) were also prepared with PCR. The biotin‐labeled and unlabeled DNA fragments of the *S. aureus hu* gene and P*agr2* served as controls. Gradient concentrations of CcpA (0–3.2 μmol) were prepared. EMSA was performed in 20 μl of gel shift loading buffer (10 mM Tris‐HCl (pH 8.0), 5 mM MgCl_2_, 1 mM DTT, 50 mM KCl, 0.05% (v/v) Nonidet® P‐40, 2.5% (v/v) glycerol) containing 50 pmol of biotin‐labeled P*crt* or P*crt*M DNA probes. A 10‐fold amount (500 pmol) of unlabeled P*agr2* DNA was included as a specific competitor, alongside an equivalent amount of *hu* DNA fragment as a nonspecific competitor. After incubation at 25°C for 30 min, 5 μl of gel loading buffer (5×) was added, and the mixture was electrophoresed in a 6% (m/v) native polyacrylamide gel in 0.5× Tris/Borate/EDTA (TBE) buffer before transferring to a nylon membrane (Millipore). Band shifts were analyzed using a LightShift Chemiluminescent EMSA Kit (Thermo Scientific), and images were captured using a ChemiDoc Touch Imaging Instrument (Bio‐Rad).

### Whole‐blood survival assay

The whole‐blood survival assay was conducted as previously reported^9^. Briefly, *S. aureus* was overnight cultivated in BHI at 37°C. Bacterial cells were harvested, washed twice with sterile PBS, and diluted to an OD_600_ value of 0.14. Approximately 5 × 10^6^ CFU in 50 µl of PBS were mixed with 450 µl of blood derived from Sprague–Dawley mice. The tubes were capped and incubated with mixing at 37°C. About 20 µl samples were taken after 1 or 2 h of incubation, serially diluted, and plated onto BHI agar plates to count CFUs after culture. The percentage of bacterial survival was calculated as (CFU_time point_/CFU_initial input_) × 100.

### Macrophage killing assay

RAW264.7 macrophages were cultivated in high‐glucose DMEM (Dulbecco's modified Eagle's medium) enriched with 10% (v/v) fetal bovine serum at 37°C in 5% (v/v) CO_2_. *S. aureus* cultures in the exponential growth phase were harvested and diluted in DMEM. The macrophages were then challenged with *S. aureus* strains of interest at a MOI of 10 in 24‐well plates. At 1 or 2 hpi, the supernatant was discarded, and cells were washed with sterile PBS. To eliminate extracellular bacteria, 1 ml of DMEM supplemented with lysostaphin (1 mg/ml) and gentamicin (50 µg/ml) was added, followed by incubation at 37°C for 1 h. Subsequently, macrophages were disrupted using PBS containing 1% (v/v) Triton X‐100, and intracellular bacterial load was counted by plating serial dilutions onto BHI agar plates and culturing overnight at 37°C. All experiments were conducted in triplicate.

### *S. aureus* growth curve

The growth kinetics of *S. aureus* strains were assessed as previously described^54^. Briefly, overnight cultures of *S. aureus* strains were grown at 37°C in BHI medium with shaking. Approximately 200 μl of culture was transferred into 20 ml of fresh BHI broth in a 50 ml sterile flask. OD_600_ values were recorded hourly for 24 h. Growth curves were generated by plotting OD_600_ readings against culture time.

### Hemolysis assay

The tube‐based hemolysis assay of *S. aureus* USA300 and its derivatives was performed as previously described^43^. In brief, *S. aureus* strains were cultured in BHI medium at 37°C for 16 h until reaching an OD_600_ of approximately 2.5. Subsequently, 100 μl of the culture supernatant was mixed with 900 μl of PBS containing 3% (v/v) rabbit red cells (Pengtong Medical, China) and incubated at 37°C for 20 min. After centrifugation at 1000*g* for 10 min at 4°C, the OD_543_ value of the supernatant was determined. Red cells treated with ddH_2_O and PBS served as positive and negative controls, respectively. The hemolytic activity was expressed as a percentage relative to the positive control, which was set as 100% hemolysis. For plate hemolytic activity determination, overnight cultured *S. aureus* USA300 and its derivatives were diluted (1:1000) in BHI medium. A 20 μl aliquot of each sample was streaked onto a Columbia sheep blood agar plate (Pangtong Medical, China) and incubated at 37°C for 16 h. After incubation, the plate was stored at 4°C for 24 h and photographed.

### Antibiotic susceptibility detection

*S. aureus* strains were cultivated overnight in MHB medium (Oxoid) with or without STX supplementation (1.0 mg/ml). Antibiotic susceptibility was detected using E‐test strips (Liofilchem, Italy) according to the instructions of the manufacturer. Antibiotics such as cefaclor, rifampicin, teicoplanin, VAN, clindamycin, amikacin, daptomycin, and ciprofloxacin were used.

### *S. aureus* growth after treatment with VAN and/or Ag^+^

The MIC of VAN or AgNO_3_ (Ag^+^) against VISA strain XN108 was determined as previously described^54^. For bacterial survival, *S. aureus* XN108 was cultivated in 2 ml of BHI overnight with shaking at 37°C. The bacterial culture at the exponential growth phase was diluted in BHI to achieve an OD_600_ of 0.10. A total of 20 μl of bacteria was transferred into 2 ml of fresh BHI containing 4 μg/ml VAN and or 4 μg/ml AgNO_3_ and cultivated at 37°C with shaking. Samples (100 μl) were collected at 0, 2, 4, 6, and 8 h for determination of CFU per milliliter. In particular, 100 μl of 10‐fold serial dilutions in PBS were spread on BHI agar plates and colonies were counted after 24 h of growth at 37°C.

### Animal experiments

The animal experiments were performed as previously described^6^, ^43^. Briefly, female BALB/c mice that were 6–8 weeks old were obtained from Weitonglihua Co. (China), and the experiments were approved by the Laboratory Animal Welfare and Ethics Committee of the Army Medical University (No. AMUWEC20211118). The *S. aureus* strain USA300 and its derivatives were cultured overnight in BHI broth at 37°C with shaking. Next, the bacterial culture was 1:100 diluted in fresh BHI medium and grown at 37°C for 6 h to obtain an OD_600_ of 1.0. Bacterial cells were pelleted, washed twice with PBS, and adjusted to 1 × 10^7^ CFU/ml to serve as a stock.

For systemic dissemination, BALB/c mice (*n* = 8) were infected with 1 × 10^6^ CFU of USA300, USA300∆*ccpA*, or USA300∆*ccpA*/pLI*ccpA* via a tail‐vein injection. The challenged animals were killed at 5 dpi. Bacterial loads in mouse livers and kidneys were calculated using the plating method as previously described^55^. For skin abscess formation, BALB/c mice (*n* = 8) were challenged subcutaneously with 1 × 10^7^ CFU of bacteria (100 µl) or PBS. Abscesses were monitored daily by measuring their dimensions, and mouse body weights were also recorded. Skin lesions from infected mice (*n* = 2) were collected on 7 dpi, fixed in 4% paraformaldehyde, embedded in paraffin, sliced, and stained with H&E. The sections were finally observed under a microscope (Olympus BX53).

For Ag^+^ treatment, the skin abscess model was generated. VAN and AgNO_3_ were combined in cream and spread onto the abscesses two times a day. After 72 hpi, the infected mice were killed, and the local abscesses were collected and homogenized in 1 ml of PBS with 1% (v/v) Triton X‐100. The tissue solution was serially diluted with PBS and spread onto BHI plates for bacterial counting. The log_10_CFU values were calculated for plotting.

### Statistical analysis

Data analysis was conducted using GraphPad Prism 9.0. Each experiment was repeated three times. The data were presented as mean ± standard derivation (SD). The unpaired Student's *t‐*test was used to analyze significant differences between two samples, and one‐way or two‐way analysis of variance (ANOVA) was used for multiple sample comparisons. The Log‐rank test was applied to assess the intergroup significant difference in survival rates. The Mann–Whitney *U* test was performed to compare the bacterial load in the skin abscesses. A *p* value less than 0.05 was considered to indicate statistical significance.

## AUTHOR CONTRIBUTIONS

**Xian Chen:** Investigation; formal analysis; data curation. **Huagang Peng:** Data curation; investigation; validation. **Xiancai Rao:** Conceptualization; supervision; writing—review and editing. **Yi Yang:** Conceptualization; methodology. **Keting Zhu:** Methodology; formal analysis. **Zhen Hu:** Resources; methodology. **Shu Li:** Conceptualization; formal analysis. **Xiaonan Huang:** Validation; methodology. **Feng Lin:** Investigation; validation. **Jianghong Wu:** Methodology; formal analysis. **Weilong Shang**: Validation; writing—original draft; conceptualization; investigation; resources. **Renjie Zhou**: Project administration; supervision; conceptualization; writing—review and editing. **Yifan Rao:** Conceptualization; funding acquisition; writing—review and editing; writing—original draft; investigation.

## ETHICS STATEMENT

The animal experiments were approval by the Laboratory Animal Welfare and Ethics Committee of the Army Medical University (No. AMUWEC20211118).

## CONFLICT OF INTERESTS

The authors declare no conflicts of interests.

## Supporting information

250909‐Suppl M.

## Data Availability

The authors confirm that the data supporting the findings of this study are available within the article and its supporting information materials.
