# Supplementary material for: CcpA promotes Staphylococcus aureus virulence by directly controlling staphyloxanthin production
Source: mLife. 2025 Dec 25;4(6):653–65. doi: 10.1002/mlf2.70040 (PMC12754628; doi:10.1002/mlf2.70040)
Supplement: Supplementary file 1 — 250909‐Suppl M. [file MLF2-4-653-s001.docx]

**Supplementary materials**

**Supplementary Figures**


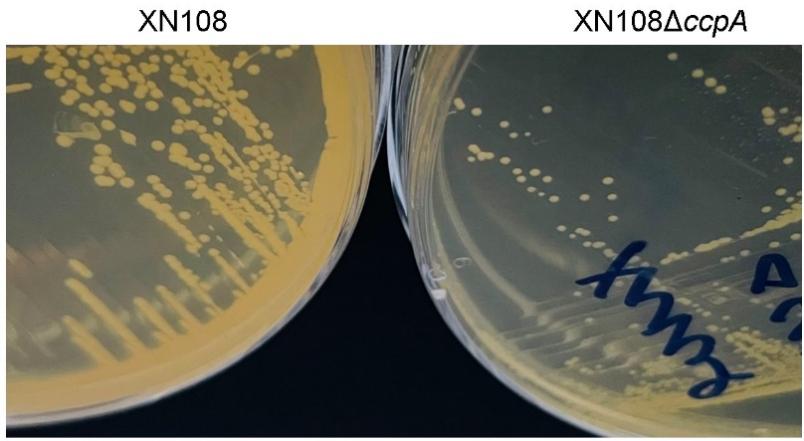


**Figure S1.** Deletion of *ccpA* decreases STX production in VISA XN108. *S. aureus* XN108 and XN108Δ*ccpA* were cultured overnight in BHI agar plates and photographed.


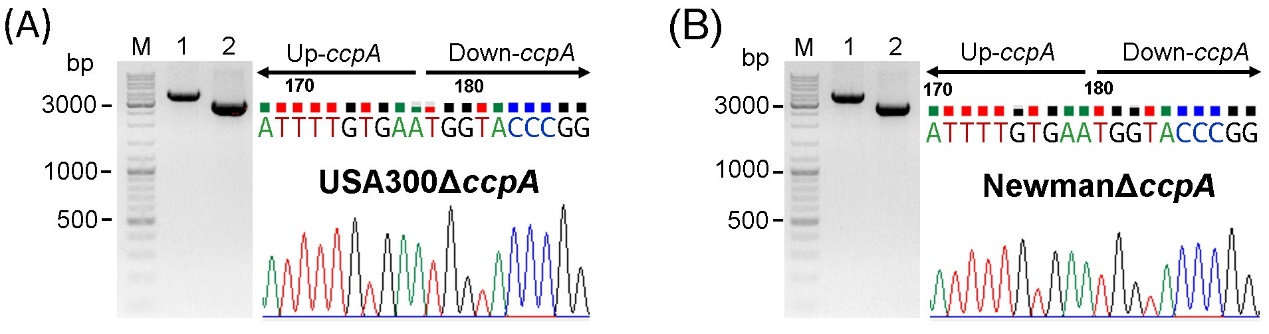


**Figure S2.** Identification of the *ccpA* deletion mutants. Characterization of USA300Δ*ccpA* (A) and NewmanΔ*ccpA* (B) by PCR (left panels) and DNA sequencing (right panels). *ccpA* deletion mutants (lane 2) exhibited relative smaller DNA products than their wide-type strains (lane 1). The sizes of the DNA ladder (M) are shown on the left.


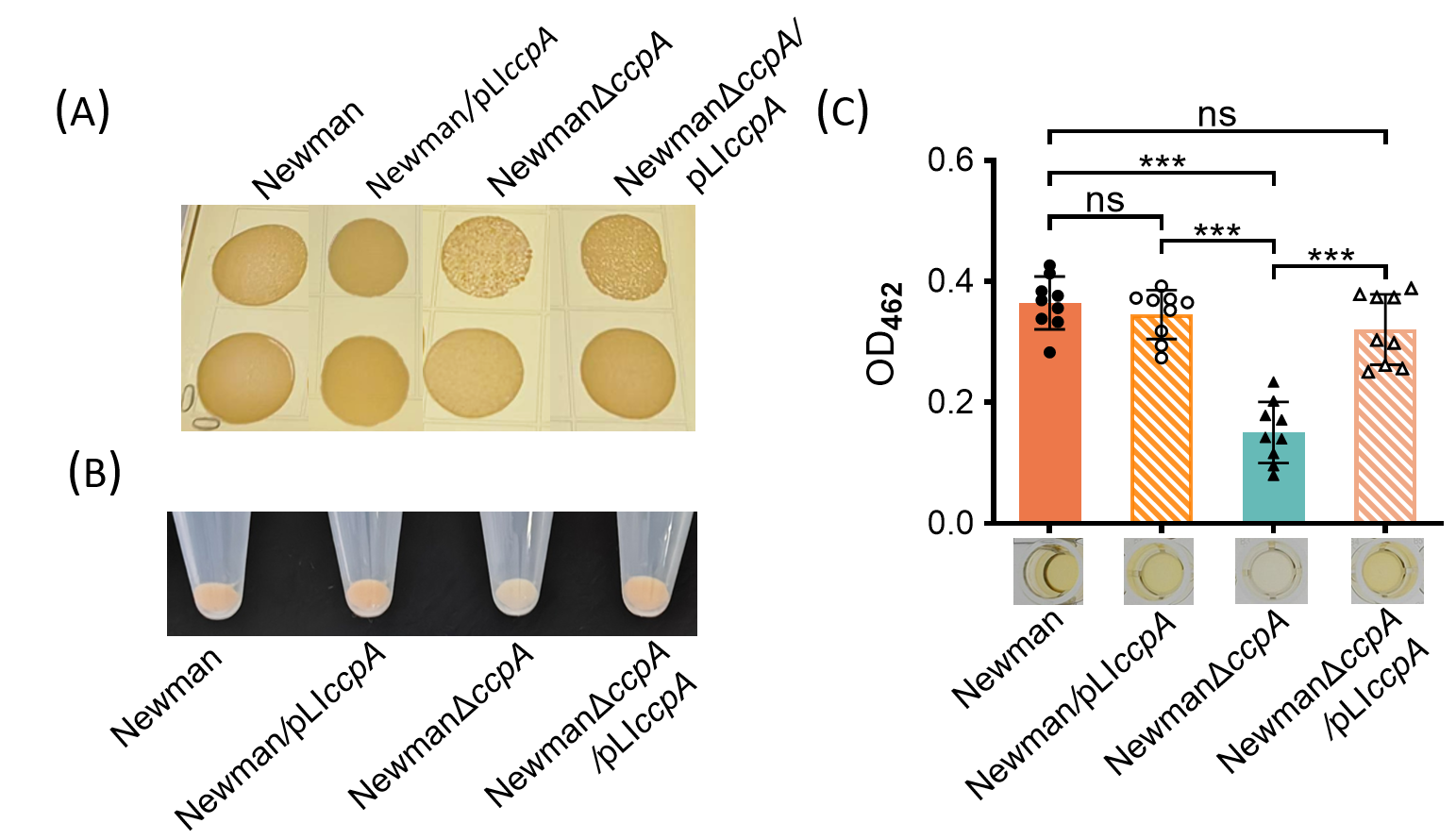


**Figure S3.** Deletion of *ccpA* decreases STX production in MSSA strain Newman. (A) Colonies of *S. aureus* Newman and its derivatives. (B) Bacterial pellets of Newman and its derivatives. (C) STX amount produced by Newman and its derivatives. Bacterial colonies were cultured and cells were pelleted. STX was extracted with methanol and quantitated by detecting OD_462_ values. The experiments were repeated three times, and data are shown as mean ± SD. The significance was calculated by one-way ANOVA. ****p* < 0.001 and ns represents no significance.


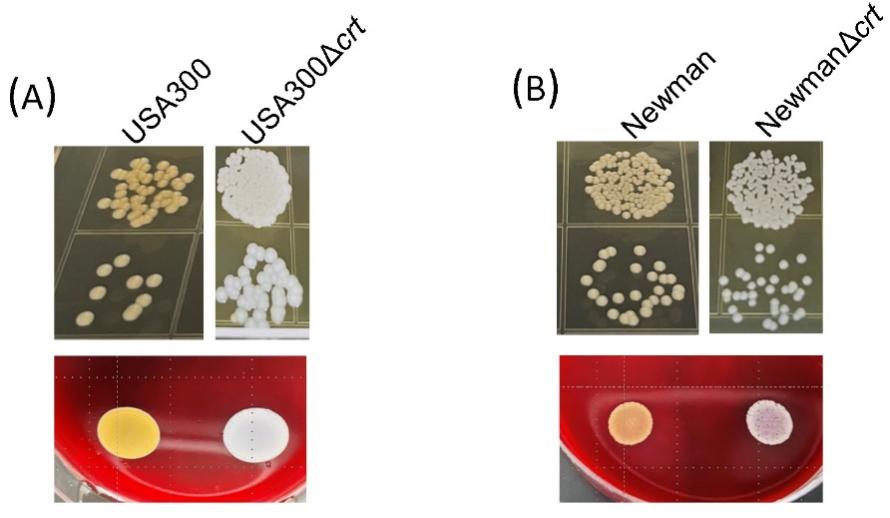


**Figure S4.** Absence of *crt* operon results in white colonies of *S. aureus*. Bacterial strains USA300 and USA300Δ*crt* (A), and Newman and NewmanΔ*crt* (B) were grown in BHI (up panels) and blood agar plates (bottom panels). After 24 h of culture at 37°C, the plates were photographed.


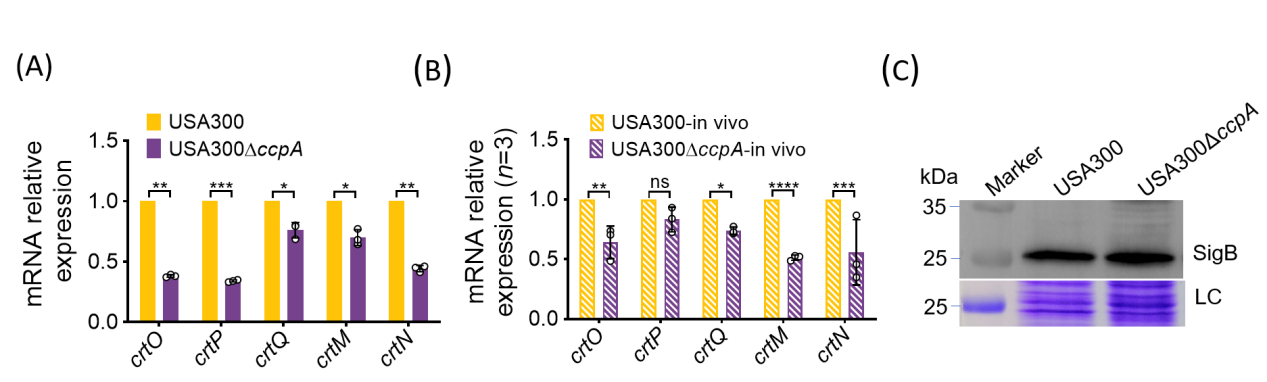


**Figure S5.** Regulation of *crt* operon by CcpA is SigB independent. (A) RT-qPCR detection of *crt* operon expression in USA300 and USA300Δ*ccpA* (A) cultured *in vitro*, and USA300 and USA300Δ*ccpA* (B) from mouse skin abscess tissues*.* The relative expression of each *crt* gene to the reference *gyrB* is presented. Statistical significance was calculated by Student’s *t*-test. **p* < 0.05, ***p* < 0.01, ****p* < 0.001, *****p* < 0.0001 and ns represents no significance. (C) Western blot analysis of SigB in USA300 and USA300Δ*ccpA* cultured *in vitro*. Molecular weights of the protein marker are indicated on the left. LC, loading control.


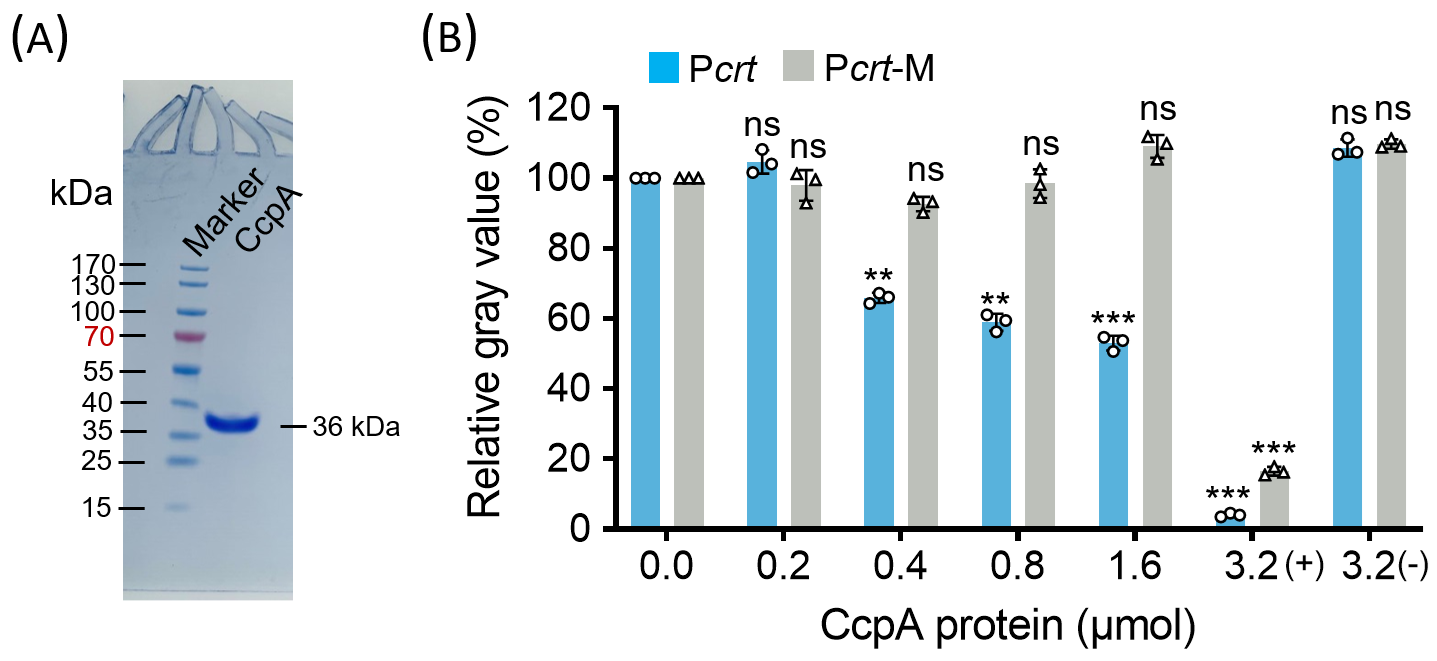


**Figure S6.** CcpA binds *crt* promoter DNA in a dose-dependent manner. (A) SDS-PAGE analysis of the purified recombinant CcpA. (B) Evaluation of gray value of the free probe in each lane of Figure 3C and 3D using ImageJ software. The free probe value in the first lane (0 µmol CcpA protein) was used as loading control (LC), and the relative gray values of the free probe to LC in other lanes were calculated and indicated. 3.2(+) and 3.2(-) indicate the positive (P*agr2*) and negative (*hu*) controls, respectively. Statistical significance of the relative gray value of each lane compared to its corresponding first lane (0 µmol CcpA protein, 100%) was measured by Student’s *t*-test. ***p* < 0.01, ****p* < 0.001, and ns represents no significance.


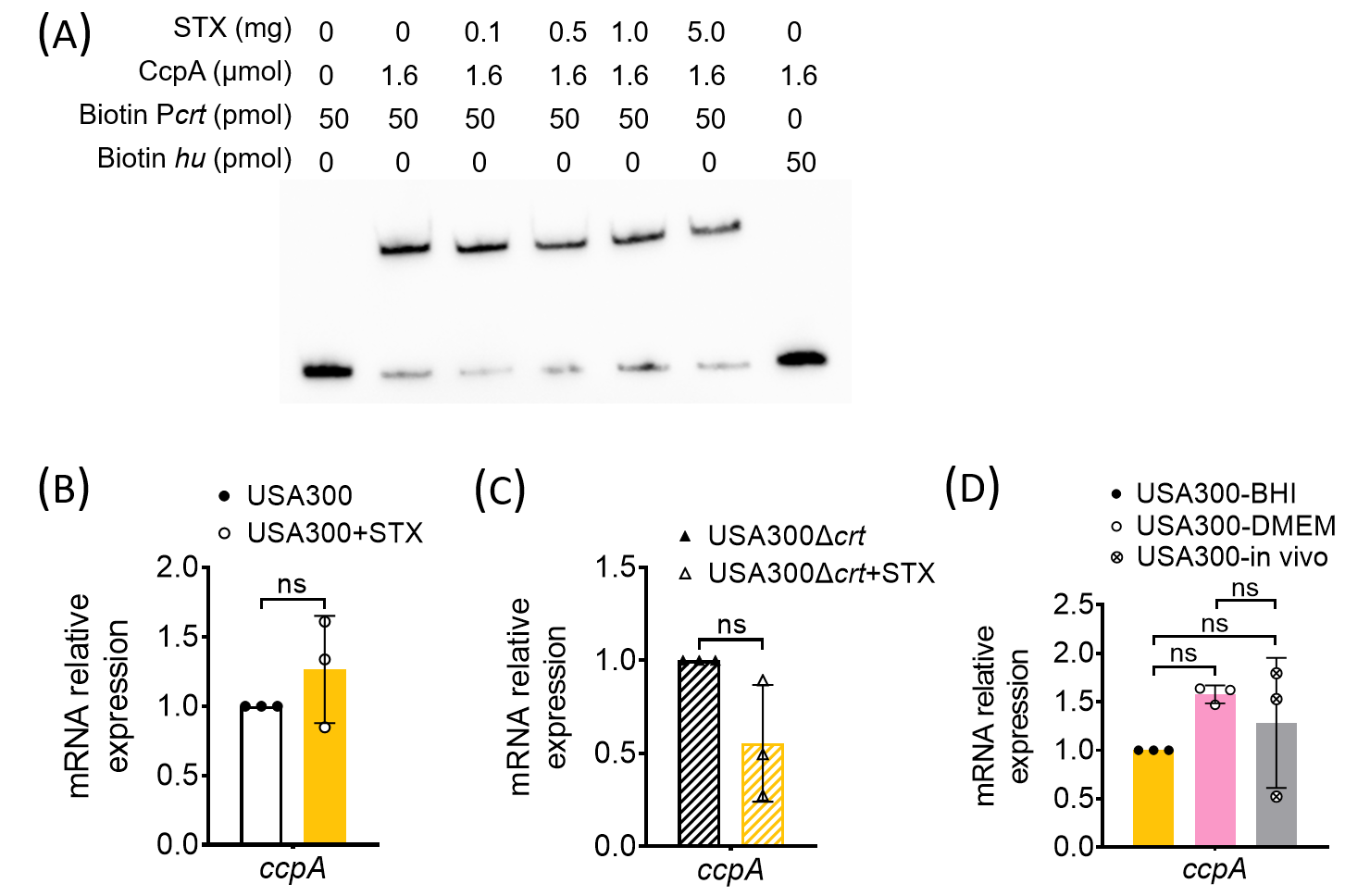


**Figure S7.** Effect of exogenous addition of STX on EMSA and *ccpA* expression. (A) STX did not affect CcpA binding to the P*crt* promoter probe. RT-qPCR detection of the *ccpA* expression levels in USA300 (B) and USA300∆*crt* (C) with or without STX treatment. (D) The expression levels of *ccpA* in USA300 cultured *in vitro* (BHI or DMEM) or infected *in vivo* (mouse liver). The relative expression of *ccpA* gene to the reference *gyrB* is presented. Data are presented as mean ± SD (*n* = 3). Statistical significance was calculated by Student’s *t*-test, and ns represents no significance.


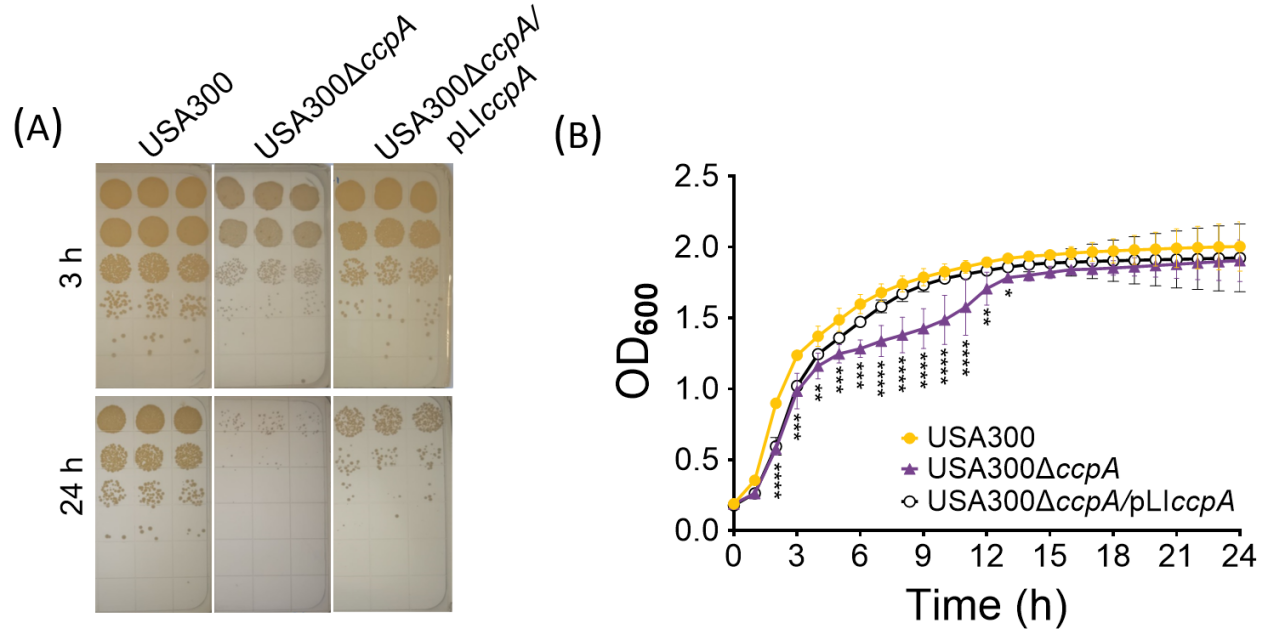


**Figure S8.** Bacterial count and growth curve. (A) Bacterial numbers by plate counting. The numbers of *S. aureus* USA300 and its derivatives in RAW264.7 macrophages after 3- and 24-h phagocytosis were determined using a plate dilution assay. (B) Growth curves of USA300 and its derivatives. The data are presented as mean ± SD of each time point. The statistical significance was calculated by two-way ANOVA. **p* < 0.05, ***p* < 0.01, ****p* < 0.001, and *****p* < 0.0001.


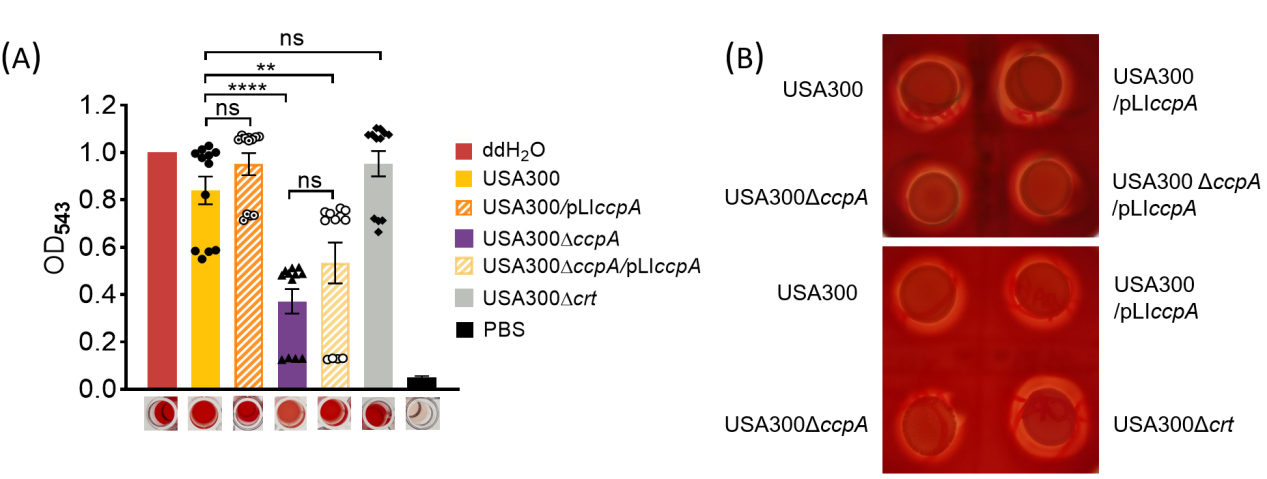


**Figure S9.** Hemolytic activities of *S. aureus* USA300 and its derivatives. (A) Relative OD_543_ values. The hemolytic activities of bacteria were detected with plate test method. The OD_543_ values of the hemolytic supernatants were determined. The OD_543_ value from each *S. aureus* strain relative to that of positive control (3% rabbit erythrocytes in 1,000 μL ddH_2_O, representing 100% hemolytic activity) was calculated and indicated. Statistical significance was calculated by One-way ANOVA. ***p* < 0.01, *****p* < 0.0001, and ns represents no significance. (B) Hemolytic activities tested with blood plates. The Columbia sheep blood agar plates were inoculated with *S. aureus* USA300 and its derivatives, and photographed after 24 h of culture.

**
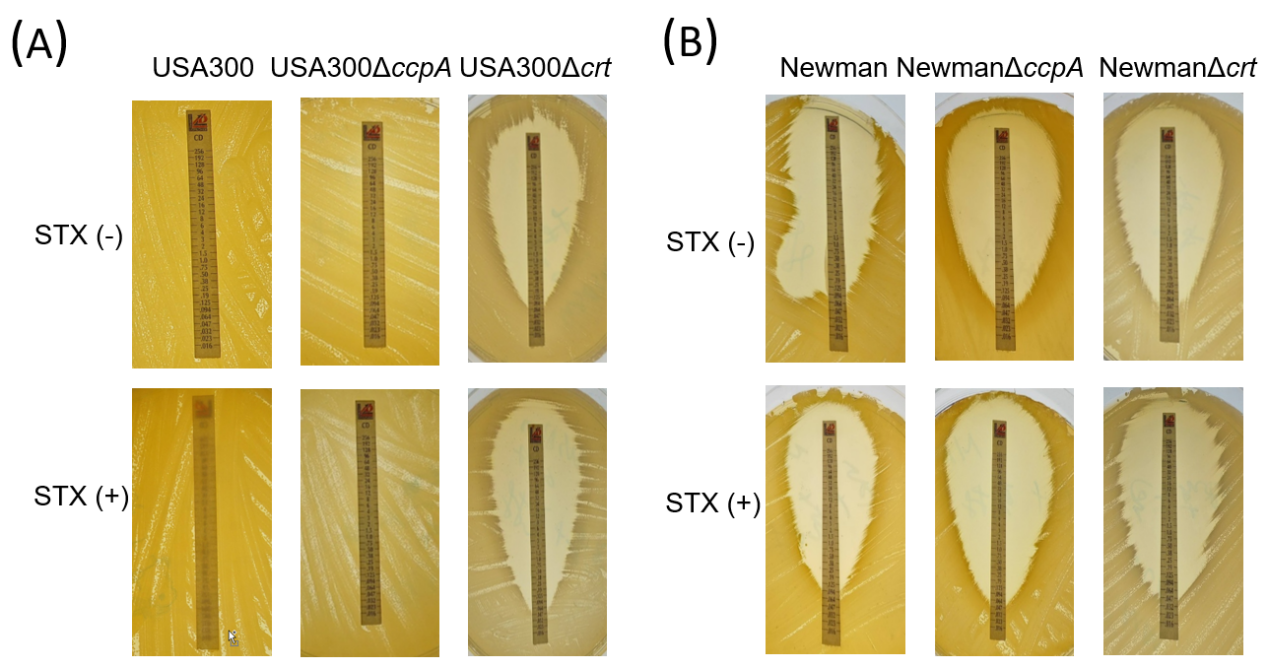
**

**Figure S10.** E-test of clindamycin susceptibilities in MRSA strain USA300 and its derivatives (A), and MSSA strain Newman and its derivatives (B) cultured in MHB medium with or without exogenous addition of STX.

**Supplementary Tables**

| ­Table S1. Strains and plasmids used in this study | | |
| --- | --- | --- |
| Strains | **Description** | **References** |
| XN108 | VISA/MRSA clinical strain with GraS(T136I), WalK(S221P), and RpoB(H481N) mutations. | [1] |
| USA300 | MRSA strain. ATCC-BAA-1556. | [2] |
| Newman | MSSA strain. NCTC 8178. | [3] |
| RN4220 | *S. aureus* strain. NCTC 8325-4. initial recipient for modification of plasmids which are introduced into *S. aureus* from *E. coli.* | [4] |
| XN108Δ*ccpA* | XN108 with *ccpA*-deleted strain. | This study |
| USA300Δ*ccpA* | USA300 with *ccpA*-deleted strain. | This study |
| NewmanΔ*ccpA* | Newman with *ccpA*-deleted strain. | This study |
| USA300Δ*ccpA/*pLI*ccpA* | Complementary strain of USA300Δ*ccpA*, Cm^r^*.* | This study |
| NewmanΔ*ccpA/*pLI*ccpA* | Complementary strain of NewmanΔ*ccpA*, Cm^r^*.* | This study |
| USA300Δ*crt* | USA300 with *crt*-deleted strain. | This study |
| NewmanΔ*crt* | Newman with *crt*-deleted strain. | This study |
| DH5α | Clone host *E. coli* strain, AMP^r^ | TransGen |
| BL21 | Protein express host *E. coli* strain (DE3), AMP^r^ | TransGen |
| Plasmids | **Description** | **Reference** |
| pBT2 | Shuttle vector, temp sensitive, AMP^r^, Cm^r^ | [5] |
| pBT2Δ*ccpA* | pBT2, for *ccpA* deletion, AMP^r^, Cm^r^ | This study |
| pBT2Δ*crt* | pBT2, for *crt* deletion, AMP^r^, Cm^r^ | This study |
| pLI50 | Shuttle cloning vector, AMP^r^, Cm^r^ | [6] |
| pLI*ccpA* | pLI50 with *ccpA* and its promoter, AMP^r^, Cm^r^ | This study |
| pOS1 | Shuttle vector, with *lacZ* ORF lacking first 6 amino acids, AMP^r^, Cm^r^ | [7] |
| pOS1-*crt*P | pOS1 derivative, harboring *crt* promoter coding sequence from strain USA300, AMP^r^, Cm^r^ | This study |
| pET28a | Expression vector with a hexahistidine tag, Kan^r^ | Novagen |
| pET28-*ccpA* | Protein CcpA expression vector with a hexahistidine tag, Kan^r^ | This study |

**References**

1. Zhang X, Hu Q, Yuan W, Shang W, Cheng H, Yuan J, et al. First report of a sequence type 239 vancomycin-intermediate *Staphylococcus aureus* isolate in Mainland China. *Diagn Microbiol Infect Dis.* 2013; 77:64–68.
2. Diep BA, Gill SR, Chang RF, Phan TH, Chen JH, Davidson MG, et al. Complete genome sequence of USA300, an epidemic clone of community-acquired meticillin-resistant *Staphylococcus aureus*. *Lancet*. 2006; 367:731–739.
3. Baba T, Bae T, Schneewind O, Takeuchi F, Hiramatsu K. Genome sequence of *Staphylococcus aureus* strain Newman and comparative analysis of staphylococcal genomes: polymorphism and evolution of two major pathogenicity islands. *J Bacteriol*. 2008; 190:300–310.
4. Berscheid A, Sass P, Weber-Lassalle K, Cheung AL, Bierbaum G. Revisiting the genomes of the *Staphylococcus aureus* strains NCTC 8325 and RN4220. *Int J Med Microbiol*. 2012; 302:84–87.
5. Brückner R. Gene replacement in *Staphylococcus carnosus* and *Staphylococcus xylosus*. *FEMS Microbiol Lett*. 1997; 151:1–8.
6. Shang W, Rao Y, Zheng Y, Yang Y, Hu Q, Hu Z, et al. β-Lactam antibiotics enhance the pathogenicity of methicillin-resistant *Staphylococcus aureus* via SarA-controlled lipoprotein-like cluster expression. *mBio*. 2019; 10: e00880-19.
7. Liu Y, Mu C, Ying X, Li W, Wu N, Dong J, et al. RNAIII activates map expression by forming an RNA-RNA complex in *Staphylococcus aureus*. *FEBS Lett*. 2011; 585:899–905.

| Table S2. Primers used in this study | | |
| --- | --- | --- |
| Primers | **Sequence (5'–3')** | **Description** |
| pBT2-*ccpA*-up-F | CCGGAATTCTAGTACAACCTAGAATTGCAAC | *ccpA* deletion |
| pBT2-*ccpA*-up-R | CGGGGTACCATTCACAAAATTAGGCATT |  |
| pBT2-*ccpA*-down-F | CGCGGATCCAATTTCCTCCTTGTAAA | *ccpA* deletion |
| pBT2-*ccpA*-down-R | GCGGTCGACACGTGGTTTATCTGCTAC |  |
| pLI50-*ccpA*-F | CGGGGTACCTTATTTTGTAGTTCCTCGGTA | *ccpA* complementation |
| pLI50-*ccpA*-R | CCGGTCGACTATTCCAAGGAAACTATAGACT |  |
| pOS1-*crt* -F | AGCCTTAAAGACGATCCGGGGTAAATCAAATGACGTCAATATTCTC | Reporter |
| pOS1-*crt* -R | TCACGACGTTGTAAAACGACTGTTTTAATATATTTTTTCATGG |  |
| *crtO*-RT-F | CATCATATTCAATAAAGGCCCTCG | RT-qPCR |
| *crtO*-RT-R | GCGTAGTAACTGCGTTAATCTCG |  |
| *crtP*-RT-F | GGCACAAAGTGGCTATTCGG | RT-qPCR |
| *crtP*-RT-R | GTAGTCTGACATTTGCTTCTTGC |  |
| *crtQ*-RT-F | TACTTCACAATCATTGCCCGTAA | RT-qPCR |
| *crtQ*-RT-R | ATAGAACCAAACAACCACAACAC |  |
| *crtM*-RT-F | TGGTGTTGCTGGTACAGTAGGTG | RT-qPCR |
| *crtM*-RT-R | CCGTTCATTGTCAAAATCTTCAC |  |
| *crtN*-RT-F | GGGCCGATGCGATAAAAGTG | RT-qPCR |
| *crtN*-RT-R | GCTGAACAAGAGTAATCTAAGTCTG |  |
| Crt-EMSA-5 | TCAAATGTAAATCAAATGACGTCAATATTC | EMSA |
| Crt-EMSA-5-Biotin | TCAAATGTAAATCAAATGACGTCAATATTC |  |
| Crt-EMSA-3 | CTGTTTTAATATATTTTTTCATGGTTTTCATCT |  |
| *hu*-EMSA-F | ATGCTTTACCAGCTTTGAATGCTG | EMSA |
| *hu*-EMSA-F-Biotin | ATGCTTTACCAGCTTTGAATGCTG |  |
| *hu*-EMSA-R | AAAAAGAAGCTGGTTCAGCAGTAG |  |
| P*agr2*-EMSA-F | ttagtgaatttgttcactgtgtcgata | EMSA |
| P*agr2*-EMSA-F-Biotin | ttagtgaatttgttcactgtgtcgata |  |
| P*agr2*-EMSA-R | gtatttaatattttaacataaaaaaatttacagt |  |
| P*crt*M-up-F | TCGCAGTGCAGCGGAATTC TGCAGAAATCCCACCTAAGCCAC | EMSA |
| P*crt*M-up-F-Biotin | TCGCAGTGCAGCGGAATTC TGCAGAAATCCCACCTAAGCCAC |  |
| P*crt*M-up-R | TGACACCGTCGAAAGGATCAGTATGATTGAGAGTGATTCAATTTAGATG |  |
| P*crt*M-down-F | TGAATCACTCTCAATCATACCTTTATTACTTAGTTCCCTAAAGATCAG | EMSA |
| P*crt*M-down-R | AGGTCGACTCTAGAGGATCC CAATAGTCAAACAGCAACAAATAACTAT |  |
| pET28a-CcpA-F | CGCGGATCCATGACAGTTACTATATATGATGT | Protein expression |
| pET28a-CcpA-R | CCGGTCGACTTATTTTGTAGTTCCTCGGT |  |

| **Table S3. E-test of eight antibiotics susceptibilities in USA300, Newman and its derivatives** | | | | |
| --- | --- | --- | --- | --- |
| **MIC of USA300 and its derivatives (μg/ml)** | | | | |
| **Antibiotics** | **STX** | **USA300** | **USA300Δ*ccpA*** | **USA300Δ*crt*** |
| **Cefaclor (CEC)** | **-** | 8 | 6 | 64 |
|  | **+** | 4 | 6 | 64 |
| **Rifampicin**  **(RIF)** | **-** | ＜0.016 | ＜0.016 | ＜0.016 |
|  | **+** | ＜0.016 | ＜0.016 | ＜0.016 |
| **Teicoplanin**  **(TEC)** | **-** | 0.75 | 0.38 | 1.0 |
|  | **+** | 0.75 | 0.38 | 0.5 |
| **Vancomycin**  **(VAN)** | **-** | 1.5 | 0.75 | 1.0 |
|  | **+** | 1.0 | 0.75 | 1.5 |
| **Clindamycin**  **(CLI)** | **-** | R | R | 0.094 |
|  | **+** | R | R | 0.064 |
| **Amikacin**  **(AMK)** | **-** | 2.5 | 1.5 | 2.0 |
|  | **+** | 3.0 | 2.0 | 2.5 |
| **Daptomycin**  **(DAP)** | **-** | 0.5 | 0.6 | 0.6 |
|  | **+** | 0.6 | 0.75 | 0.5 |
| **Ciprofloxacin**  **(CIP)** | **-** | R | R | R |
|  | **+** | R | R | R |
| **MIC of Newman and its derivatives (μg/ml)** | | | | |
| **Antibiotics** | **STX** | **Newman** | **NewmanΔ*ccpA*** | **NewmanΔ*crt*** |
| **Cefaclor (CEC)** | **-** | 1.0 | 2.5 | 1.5 |
|  | **+** | 2.0 | 1.5 | 1.5 |
| **Rifampicin**  **(RIF)** | **-** | ＜0.016 | ＜0.016 | ＜0.016 |
|  | **+** | ＜0.016 | ＜0.016 | ＜0.016 |
| **Teicoplanin**  **(TEC)** | **-** | 1.0 | 0.75 | 0.75 |
|  | **+** | 1.0 | 0.38 | 0.75 |
| **Vancomycin**  **（VAN）** | **-** | 2.0 | 1.0 | 0.75 |
|  | **+** | 1.0 | 1.0 | 1.0 |
| **Clindamycin**  **(CLI)** | **-** | 0.094 | 0.064 | 0.047 |
|  | **+** | 0.097 | 0.047 | 0.047 |
| **Amikacin**  **(AMK)** | **-** | 4 | 2 | 3 |
|  | **+** | 6 | 2 | 4 |
| **Daptomycin**  **(DAP)** | **-** | 0.75 | 0.38 | 0.5 |
|  | **+** | 1.0 | 0.75 | 0.75 |
| **Ciprofloxacin**  **(CIP)** | **-** | 0.19 | 0.125 | 0.19 |
|  | **+** | 0.19 | 0.10 | 0.19 |
